# Albumin Antioxidant Response to Stress in Diabetic Nephropathy Progression

**DOI:** 10.1371/journal.pone.0106490

**Published:** 2014-09-04

**Authors:** Rafael Medina-Navarro, Itzia Corona-Candelas, Saúl Barajas-González, Margarita Díaz-Flores, Genoveva Durán-Reyes

**Affiliations:** 1 Department of Experimental Metabolism, Center for Biomedical Research of Michoacán (CIBIMI-IMSS), Morelia, Michoacán, México; 2 Department of Nephrology, General Regional Hospital N° 1, IMSS, Morelia, Michoacán, Mexico; 3 Biochemistry Medical Research Unit, National Medical Center, IMSS, México City, México; Fondazione IRCCS Ospedale Maggiore Policlinico & Fondazione D’Amico per la Ricerca sulle Malattie Renali, Italy

## Abstract

**Background:**

A new component of the protein antioxidant capacity, designated Response Surplus (RS), was recently described. A major feature of this component is the close relationship between protein antioxidant capacity and molecular structure. Oxidative stress is associated with renal dysfunction in patients with renal failure, and plasma albumin is the target of massive oxidation in nephrotic syndrome and diabetic nephropathy. The aim of the present study was to explore the albumin redox state and the RS component of human albumin isolated from diabetic patients with progressive renal damage.

**Methods/Principal Findings:**

Serum aliquots were collected and albumin isolated from 125 diabetic patients divided into 5 groups according to their estimated glomerular filtration rate (GFR). In addition to clinical and biochemical variables, the albumin redox state, including antioxidant capacity, thiol group content, and RS component, were evaluated. The albumin antioxidant capacity and thiol group content were reciprocally related to the RS component in association with GFR reduction. The GFR decline and RS component were significantly negatively correlated (R = –0.83, p<0.0001). Age, creatinine, thiol groups, and antioxidant capacity were also significantly related to the GFR decline (R = –0.47, p<0.001; R = –0.68, p<0.0001; R = 0.44, p<0.001; and R = 0.72, p<0.0001).

**Conclusion/Significance:**

The response of human albumin to stress in relation to the progression of diabetic renal disease was evaluated. The findings confirm that the albumin molecular structure is closely related to its redox state, and is a key factor in the progression of diabetes nephropathy.

## Introduction

Increasing evidence supports an association between the serum albumin concentration and mortality from several diseases [1]–[4]. The integrity of the albumin molecule might be a key determinant of its biologic activity [5], [6]. Structural stress induced by nonenzymatic glycation or reactive oxygen species impairs the antioxidant capabilities of albumin, a factor associated with the development of diabetes complications [7]. Evidence of protein stress (carbonyl content, dityrosine content) is detected in patients with diabetic nephropathy (DN) [8], [9]. How this stress affects the protein antioxidant response and the molecular structure of albumin during the progression of DN, however, is not clear. Albumin has antioxidant properties [10] and is the major antioxidant in plasma, which is continuously exposed to oxidative stress [11].

A new component of the protein antioxidant capacity (AC) was recently described [12]. The intrinsic component, designated Response Surplus (RS), represents the antioxidant response that occurs when proteins undergo a structural perturbation by a stressor, such as temperature, short-wave ultraviolet (UV) light, and reactive oxygen species. The change in the AC of proteins is closely related to their molecular structure [12].

Previous studies demonstrated the impaired antioxidant capacity of albumin as a consequence of various structural modifications [13], [14]. The changes were clearly linked to the passive redox state of the thiol groups and particularly to the albumin redox active thiol group (Cys-34). Therefore, the AC of any biologic system is very complex, and the albumin Cys-34-dependent AC of albumin represents a passive component, whereas the RS represents an active component linked to changes in the molecular structure [12].

**Figure 1:**
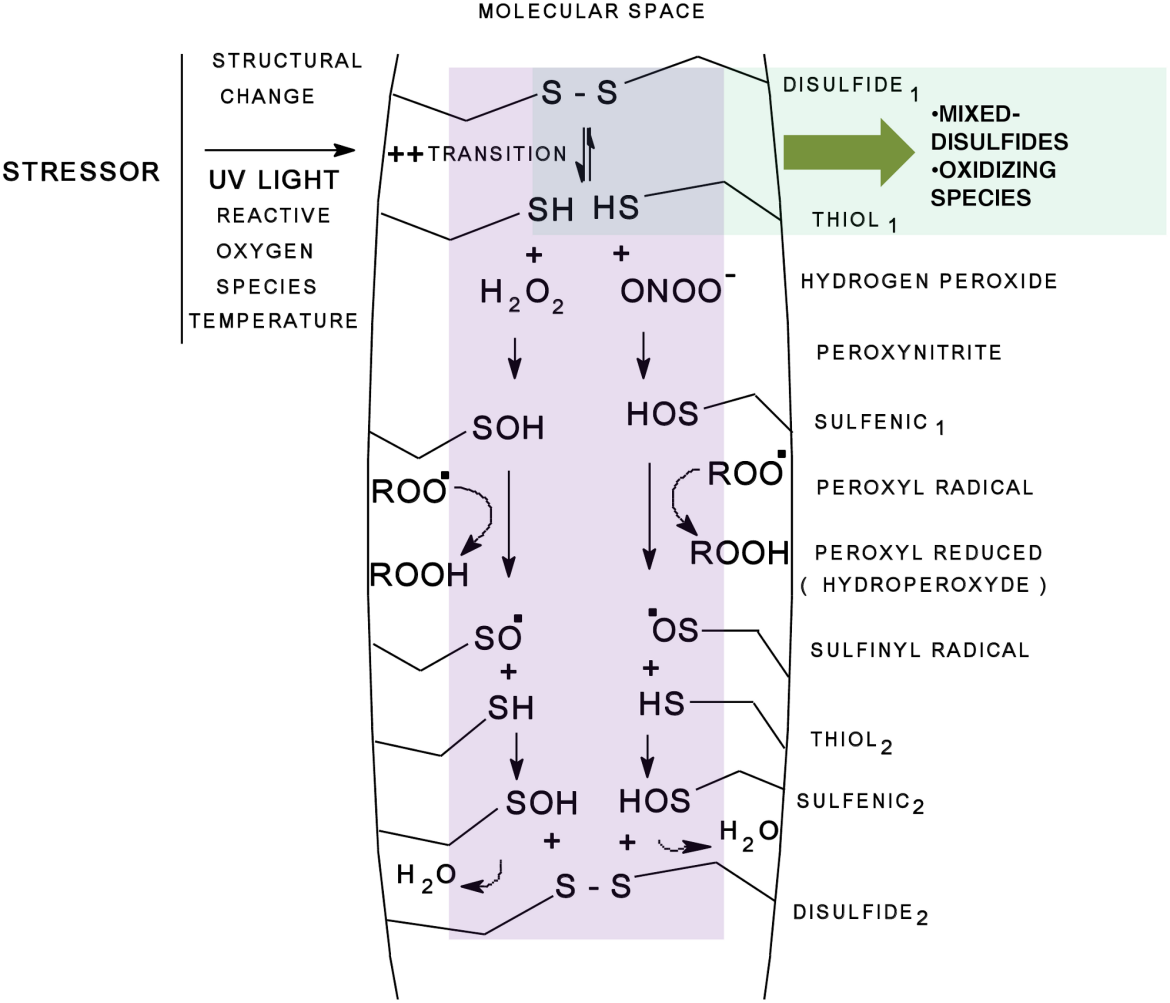
In accordance with the model proposed to explain the Response Surplus (RS), a stressor introduces instability into the core of a protein, which promotes the transition (structural perturbation) between the disulfide and thiol (SH) groups. The SH groups then interact with reactive oxygen species and form mixed disulfides. Although it is a primer, it is part of the passive antioxidant capacity of a protein and is Cys34-dependent. In contrast, in a second dynamic phase, a sulfenic acid derivative forms from the reaction between reactive oxygen species and SH groups can reduce free radicals such as peroxyl radicals (ROO) with high efficiency, as previously reported [15], generating sulfinyl radical derivatives (RSO) as the product of the reaction. Then, a second stock of sulfenic acid can be recycled from RSO if enough SHs are available, which will be converted to a new S-S group. From this model, we can conclude that the Cys34 chemistry provides only a limited explanation and cannot account for how albumin functions as an antioxidant in an oxidative milieu; the RS component represents a possible explanation.

The model proposed to explain the RS is presented in Figure 1. Using this model, we aim to explain the mechanism underlying the albumin antioxidant response to stress that can only be partially accounted for by the Cys34 chemistry. The RS component represents a measure of the response to this stress.

In the present work, we determined for the first time the RS component of human serum albumin (HSA) isolated from diabetic patients with progressive renal damage. In addition, the AC and evidence of structural alterations of the albumin molecule were determined using fluorescence spectroscopy to measure hydrophobicity and intrinsic fluorescence and quenching. The results demonstrated a reciprocal progressive reduction of the AC, progression of the RS component, and evidence of structural changes, all of which correlated with the progressive stages of DN.

## Results and Discussion

### Patient characteristics, biochemistry, and redox variables

The principal clinical and biochemical differences between patients in Stages 1–5 DN compared with the Stage 0 control group are presented in Table 1. As expected, together with the estimated glomerular filtration rate (GFR), the glycated hemoglobin (HbA1c), blood creatinine levels, and age differed between all stages and the control group; differences were greater in patients in advanced stages of DN. Age was not significantly different between Stage 1 and Stage 0. Mean body mass index (BMI) in patients from Stages 1–5 was 27.8 Kg m^2^, lower than the mean value of the control group; however, the BMI of the Stage 1 group was significantly higher than that in all other groups, including the control group (33.6±6.1). Mean HbA1c was 7.1% and significantly differed between Stages 1–5 and the control group; however, there were no differences among Stage 1–5 groups. The corresponding BMI indicated that all groups except Stage 5 were overweight; female sex predominated in Stages 1–3, and male sex predominated in Stage 5. Additional information regarding the medications prescribed for patients included in the study, some of which are associated with comorbidities, is included in (Table S1). Additional information regarding variables Stages 1–5 and Control in (File S1).

**Table 1:** Characteristics and differences between the patients grouped by aGFR (Stages 0–5).

Characteristics	CONTROL(Stage 0)	Stage 1	Stage 2	Stage 3	Stage 4	Stage 5
		GFR≥90	GFR 60–89	GFR 30–59	GFR 15–29	GFR*<*15
n (male:female)	20(7∶13)	25(7∶18)	25(6∶19)	25(8∶17)	25(12∶13)	25(16∶9)
Age (years)	49±8	48±8	58±6**	62±6***	60±9**	61±7***
bBMI (kg m^−2^)	28.3±11	33.6±6.1*	28.2±4.9	27.3±5.1	26.2±6.1	23.7±9.3
cHb_A1c_ (%)	6.2±0.60	7.34±1.3*	7.1±1.3*	7.1±0.9*	7.1±0.2*	7.2±0.9*
Blood Creatinine(mg dl^−1^)	0.75±0.18	0.79±0.09**	0.98±0.11**	1.02±0.11***	3.94±0.94***	8.9±2.46***
Serum Albumin(mg/dl)	5.7±1.8	4.7±2.1	4.0±0.4***	4.0±0.5***	3.7±0.5***	3.6±0.5***
GFR(mL min^−1^ 1.73 m^−2^)	122.2±46	101.0±9.4*	74.0±6.0***	50.2±8.8***	18.53±4.3***	5.12±1.9***

a glomerular filtration rate (mL min^−1^ 1.73 m^−2^);

b body mass index;

c glycated hemoglobin.

TEU, Trolox equivalent units; RS, response surplus.

*P<0.05 vs. control; **P<0.01 vs. control; ***p<0.001 vs. Control.

Table 2 summarizes the biochemical results with respect to the redox state and RS of albumin between groups. A progressive increase in the RS% component correlated with the DN stage; from Stage 2, the differences were statistically significant (p<0.001). The differences between the Stage 1 group and the Stage 0 group were not significant. A significant difference in the concentration of thiol groups was identified in the albumin of patients from Stages 4 and 5 compared with the Stage 0 group (p<0.05), and the AC tended to decrease with significant differences between Stages 3 and 4 compared to the Stage 0 group (p<0.05) and Stage 5 with respect to the Stage 0 group (p<0.01).

**Table 2:** Redox state and RS of purified albumin between the patients grouped by aGFR (Stages 0–5).

Characteristics	CONTROL(Stage 0)	Stage 1	Stage 2	Stage 3	Stage 4	Stage 5
		GFR≥90	GFR 60–89	GFR 30–59	GFR 15–29	GFR*<*15
Thiol groups (SHs)(*µ*mol g^−1^ protein)	4.71±1.5	5.18±1.77	4.92±1.39	4.66±1.69	3.63±0.87*	3.28±1.21*
Antioxidant Capacity(bTEU nM)	7.72±1.99	7.94±0.29	6.94±0.58	6.48±0.62*	6.34±0.50*	6.04±0.54**
RS^c^ (%)	11.0±8.3	14.5±5.4	30.0±8.3***	51.9±10.8***	60.5±10.2***	74.7±11.2***

*P<0.05 vs. Control;

**P<0.01 vs. Control;

***p<0.001 vs. Control.

a glomerular filtration rate (mL min^−1^ 1.73 m^−2^);

b TEU, Trolox equivalent units;

c RS, response surplus.

The relationship between the GFR and clinical variables is shown in Table 3 . Age was significantly negatively correlated with GFR (R = –0.47, p<0.001). Creatinine was significantly correlated with the GFR (R = –0.68, p<0.0001); similarly, although with less significance, serum albumin and sex (female) correlated with the GFR (R = 0.147, p<0.043 and R = 0.195, p<0.011 respectively).

**Table 3:** Correlation between glomerular filtration rate (GFR) with principal clinical variables.

	GFR	Age	BMI^a^	Hb_A1c_^b^	Creatinine	Serum albumin	Sex
	(ml min^−1^ 1.73 m^−2^)	(years)	(kg m^−2^)	(*%*)	(mg/dl)	(mg/dl)	(0:M, 1:F)
**Range**	**3.2–134**	**28–75**	**15.5–52.8**	**5.0–8.9**	**0.7–17.0**	**2.0–9.71**	
**Pearson R**	**1.00**	**–0.47**	**0.371**	**0.028**	**–0.68**	**0.147**	**0.195**
p value	<0.0001	<0.001	<0.001	0.376	<0.0001	0.043	0.011

a body mass index; ^b^glycated hemoglobin.

The relationship between the GFR and the redox variables and RS of albumin is shown in Table 4 . Between the redox variables, the thiol groups (SHs) were significantly positively correlated with GFR (R = 0.44, p<0.001). The AC was also significantly positively correlated with GFR (R = 0.72, p<0.0001). Finally, the response to stress or RS was very strongly significantly negatively correlated with GFR (R = –0.83, p<0.0001).

**Table 4:** Correlation between GFR^a^ with redox variables and RS.

	GFR	Albumin Thiol Groups	Albumin AC^b^	Albumin RS^c^
	(mL min^−1^ 1.73 m^−2^)	(*µ*mol g^−1^ protein)	(TEU nM)	(%)
**Range**	**3.2–134**	**1.42–7.78**	**5.51–8.41**	**1.27–16.02**
**Pearson R**	**1.00**	**0.44**	**0.72**	**–0.83**
p value	<0.000	<0.001	<0.0001	<0.0001

a glomerular filtration rate (mL min^−1^ 1.73 m^−2^).

b TEU, Trolox equivalent units.

c RS, response surplus.

The factors associated with renal function decline that strongly correlated with GFR were included in a multivariate regression analyses to test the association of the variable with renal function decline (Table 5). Sex, serum albumin, HbA1c, AC, and thiol groups were excluded from the final model. RS, creatinine, and variables that significantly correlated with GFR in the bivariate analyses were tested in stepwise linear regression analyses. GFR was inversely associated with RS, creatinine, and age, and positively associated with BMI, all variables accounting for 74% of the variability in the GFR (Table 5). These findings indicate that the redox state of albumin is highly related with DN and the decline of renal function. Although the antioxidant properties of HSA are largely attributed to the Cys34 chemistry and the ratio of the albumin forms mercaptoalbumin and non-mercaptolbumin [16], the results obtained here and previously [12] suggest that additional mechanisms tightly linked with the molecular structure are involved in the redox balance and albumin antioxidant properties. Interestingly, even with a high correlation, the redox components AC and thiol groups were excluded from the final regression analyses model in contrast with the strong association of RS, which suggests that RS is related with the GFR through pathways other than oxidative stress and possibly related with albumin structural changes.

**Table 5:** Stepwise linear regression models for parameters associated with GFR.

Independentvariable	RegressionCoefficient (95% CI)	StandarizedRegressionCoefficient	P
**Model 1**			
RS	–1.537 (–1.709, –1.364)	0.834	<0.0001
**Model 2**			
RS	–1.317 (–1.524, –1.111)	–0.715	<0.0001
Creatinine	–2.666 (–4.159, –1.173)	–0.200	0.001
**Model 3**			
RS	–1.168 (–1.387, –0.949)	–0.634	<0.0001
Creatinine	–2.825 (–4.271, –1.379)	–0.212	<0.0001
Age	–0.773 (–1.244, –0.303)	–0.161	0.001
**Model 4**			
RS	–1.126 (–1.344, –0.907)	–0.611	<0.0001
Creatinine	–2.649 (–4.080, –1.219)	–0.199	<0.0001
Age	–0.754 (–1.218, –0.291)	–0.157	0.002
BMI	0.625 (0.96, 1.158)	0.107	0.021
**Excluded**			
Sex	–0.028		0.534
Serum albumin	0.032		0.462
AC	–0.002		0.973
Thiol groups	–0.001		0.988

RS, response surplus; BMI, body mass index; AC, antioxidant capacity; GFR, glomerular filtration rate. Only variables that significantly correlated with the dependent variable GFR in the bivariate analyses were tested in stepwise linear regression analyses. GFR was inversely associated with RS, creatinine, and age, and positively associated with BMI; all variables accounting for 74% of the variability in the GFR.

### The native albumin response to short-wave UV light as a stressor

**Figure 2:**
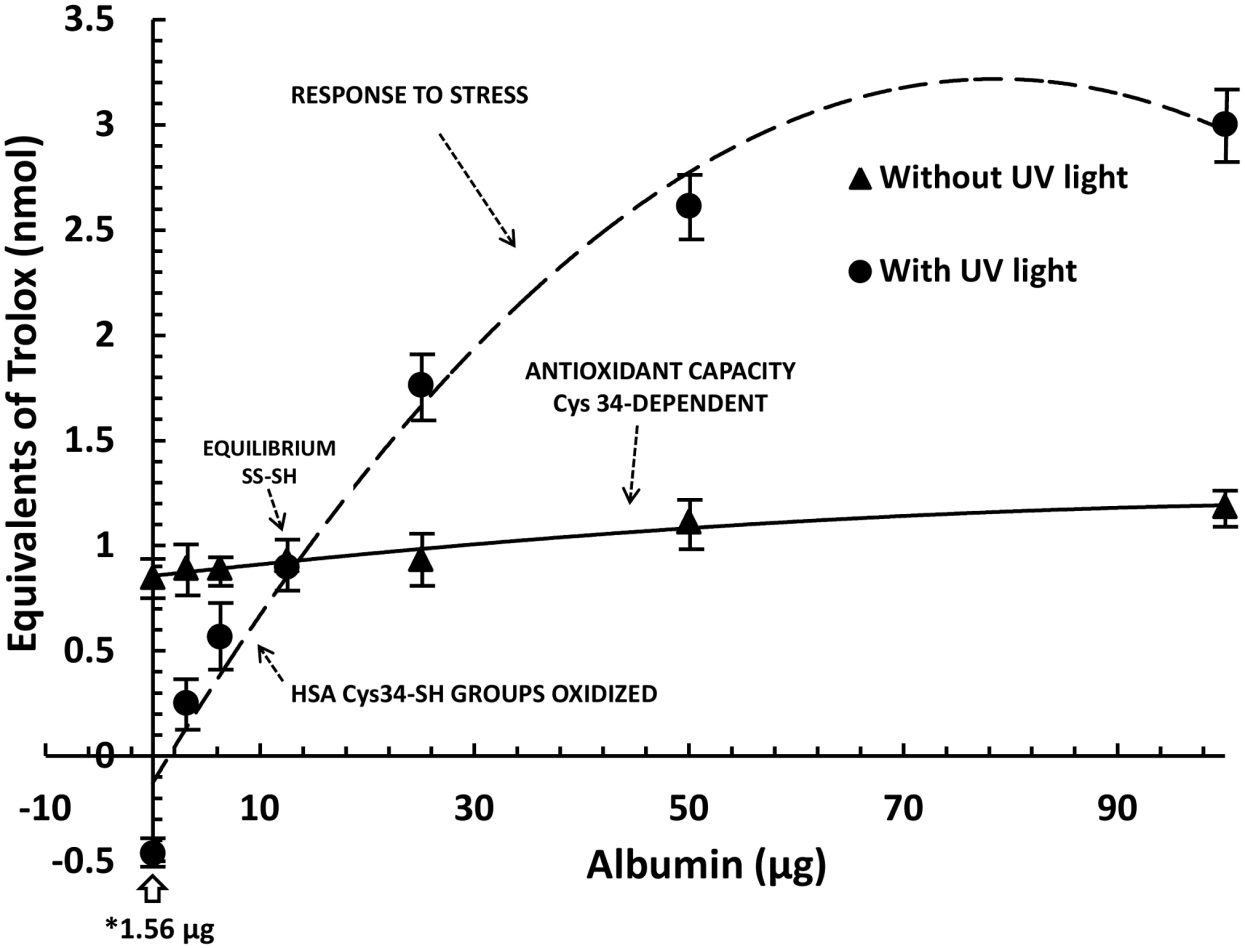
Native albumin antioxidant response to stress produced by short-wave UV light. Triangles (p) represent the antioxidant capacity (AC) of native serum albumin in relation to protein concentration. Circles (∼) represent the antioxidant capacity of the increased amounts of native albumin previously exposed to UV light (254 nm) in the conditions described in the Materials and Methods. Results are expressed as mean ± SD. The light energy was calculated at 10 mW cm^−2^; * 1.56 µg corresponds to the minimal amount of protein. The AC of non-stressed albumin has a concentration–dependent linear relationship. In stressed albumin, however, the AC has a biphasic in relation to the albumin concentration. Beginning at a concentration of 12.5 µg (SS-SH equilibrium), the AC progressively increases to 1-, 2-, and 3-fold the basal amount in stressed albumin in a concentration-dependent manner (1.76, 2.61, and 3.0 equivalents of Trolox (nmol) with 25, 50, and 100 µg of protein, respectively. At a low concentration, the human serum albumin (HSA) Cys34-SH groups are oxidized and the AC decreases. At a specific protein concentration (12.5 µg), a balance can be observed between albumin oxidation and thiolation (reversible states of oxidation). In stressed conditions at above12.5 µg, the AC could not be produced exclusively from the Cys34 SH and intermediates, and there is a loss of linearity; the point at which the Response Surplus is produced.

The antioxidant response of proteins to several stressors has been described [12]. In the present work, short-wave UV light (245 nm) was used as the stressor, because the results are particularly clean and reproducible. The energy of the light used in the experiments was calculated at 10 mW cm^−2^ and the samples were exposed to UV radiation as described in Materials and Methods. The change in the antioxidant potential in relation to the protein concentration is shown in Figure 2 , and corresponds to commercial delipidated albumin with and without exposure to UV light. Under normal conditions and without a stress pulse, the AC of native albumin is solely protein-concentration dependent, and a linear relationship is established between AC and the protein concentration (Figure 2, without UV light). In contrast, in response to a stressor, the albumin redox behavior is quite different and it is biphasic in relation with the protein amount (Figure 2, with UV light). With a low concentration of protein, the AC was lower than that of albumin without a stressor (1.56 µg is the minimal amount of albumin assayed). From this point, the AC of the albumin is increased in relation to the concentration, remaining lower than albumin without UV light up to 12.5 µg protein, a point at which it is not possible to observe differences in AC with or without a stressor. From here, the AC increases progressively to 1-, 2-, and 3-fold the basal amount in the stressed albumin in a concentration-dependent manner (1.76, 2.61, and 3.0 equivalents of Trolox [nmol] with 25, 50, and 100 µg of protein, respectively [p<0.001]).

The information gained from these experiments provides important clues to understanding the nature of the RS mechanism. In the experimental condition assays, with a low protein concentration, the oxidative stress produced by UV light oxidized all of the Cys34 SH groups and then the AC decreased. With a specific protein concentration (12.5 µg), a balance can be observed between albumin oxidation and thiolation (reversible states of oxidation) with and without stressor effects. Under stressed conditions, beginning at 12.5 µg the AC observed is not produced exclusively from the Cys34 SH and intermediates, and the loss of linearity becomes evident; a positive response to the stress becomes evident as well (Figure 2).

Under physiologic conditions and in the extracellular environment, HSA exists in two different forms, human mercaptoalbumin (HSA-reduced) with the thiol group in a reduced form, and human reversible non-mercaptoalbumin (HSA-oxidized), which presents as several kinds of mixed disulfides, with cysteine, cysteinylglycine, glutathione, homocysteine, and γ-glutamylcysteine [17], [18]. Additionally, HSA can comprise varying amounts (1%–2%) of strongly oxidized human non-mercaptoalbumin, and sulfinic and sulfonic acid (HSA-SO_2_H and HSA-SO_3_H). The reduced and oxidized forms of HSA are in dynamic equilibrium, depending on the redox state of Cys34 and the thiol disulfide exchange, patient age, and disease state [16].

The differences in AC between the stressed and non-stressed albumin presented here cannot be explained by the thiol disulfide exchange (Figure 2), particularly in a system without low molecular-weight thiols. Based on the experiments described, we confirmed the existence of an active component that was determined by changes in the tertiary structure of the protein. We isolated and studied albumin from diabetic patients at different stages of DN, looking for evidence of changes in AC and the RS component that correlated with the conformational changes in albumin and the progression of the disease.

### Protein antioxidant capacity, response surplus, and the progression of DN

**Figure 3:**
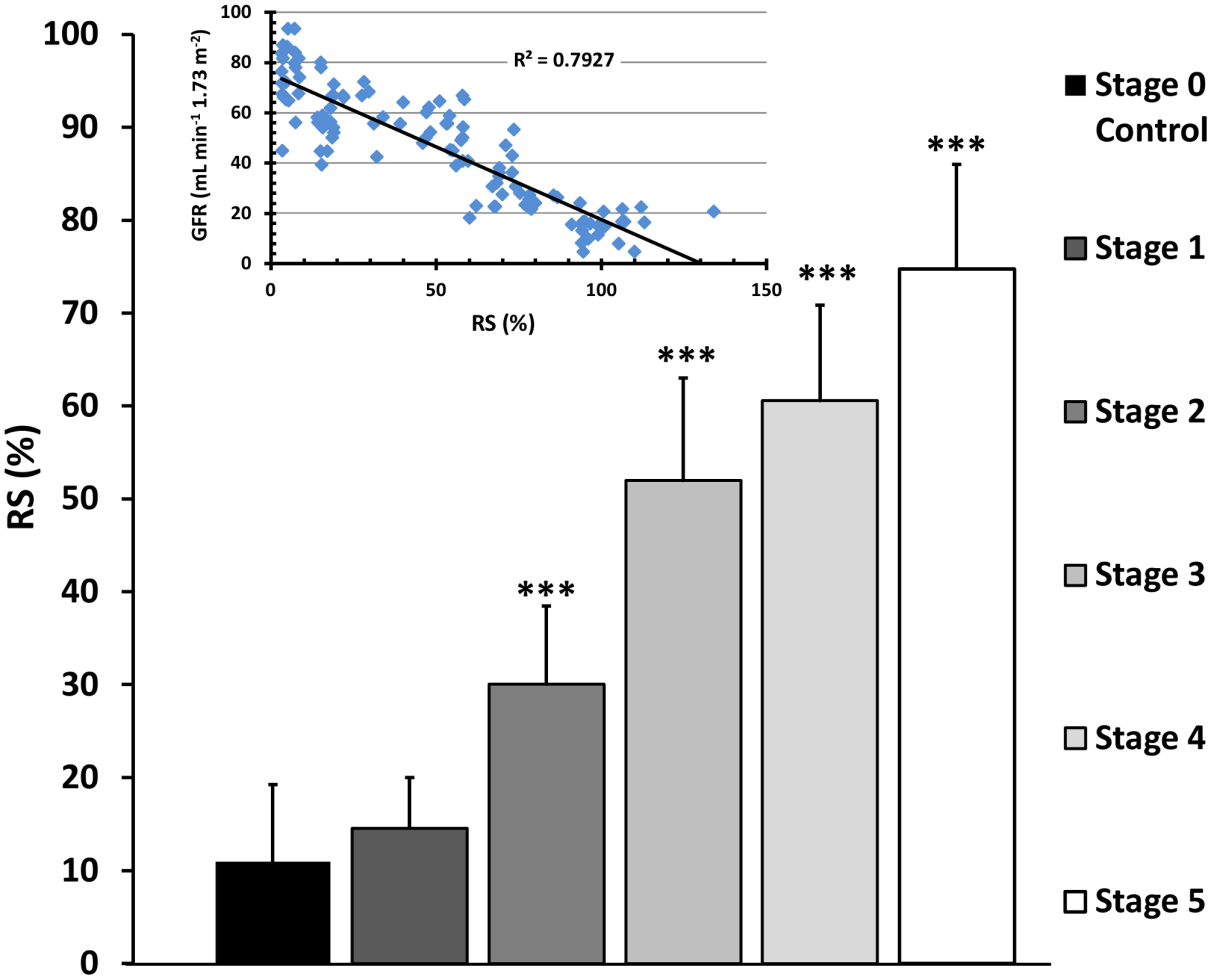
Albumin Response Surplus (RS) in the diabetic nephropathy progression. Albumin from the Stage 0 control group and patients with different stages of renal disease were isolated and the response to stress was measured as described in the Materials and Methods. The glomerular filtration rate (GFR) was highly correlated with the response surplus (RS) (internal graph, R^2^ = 0.7927). The patients were grouped as Stage 0 (control) and Stages 1–5 depending of their GFR; with the exception of Stage 1, patients in all stages showed a progressive increase in RS with a high level of significance (p<0.001). Results are expressed as mean + SD. ***P<0.001 vs. Control group.

**Figure 4:**
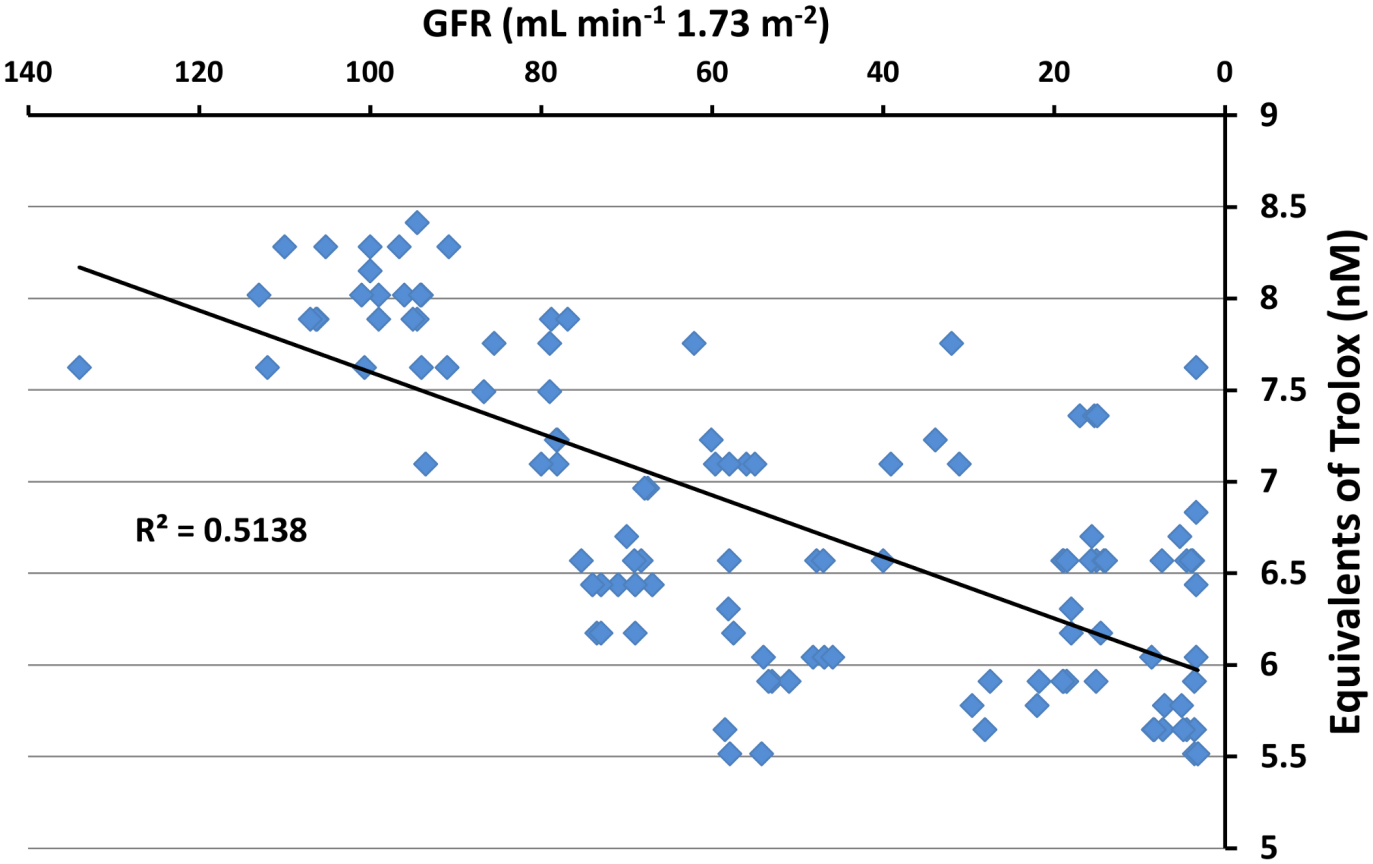
Antioxidant capacity (AC) measurement in relation to the glomerular filtration rate (GFR) of patients at different stages of diabetic nephropathy. The AC was calculated in Trolox Equivalent Units (nM) as described in the Materials and Methods section. A progressive and statistically significant correlation was observed between AC and the decline of renal function. R^2^ = coefficient of determination.

In the present study, we established a positive correlation between the DN stage and the antioxidant response to stress (RS; R^2^ = 0.7927) (Figure 3). The results demonstrated that albumin isolated and purified from diabetic patients with advanced stages of renal damage and lower GFR has a higher RS value than albumin from normal patients or patients in earlier stages of DN. In diabetes, the structural changes induced by nonenzymatic glycation or by reactive oxygen species impair the antioxidant capabilities of albumin; the stress imposed by the former changes represent the possible stressors associated with the induction of an increased response to stress (RS). In this respect, the progressive reduction in the albumin AC observed in the present study (Figure 4) accompanied by the progressive reduction in the GFR greatly support the notion that changes in the native albumin structure are associated with the loss of protein function. The postulated tight relationship between the AC and the protein conformation [12] should explain the higher response to stress as a function of the higher instability of the albumin molecule in the advanced stages of renal damage. The RS represents an indirect measure of these structural changes.

### Fluorescence spectroscopy and quenching

**Figure 5:**
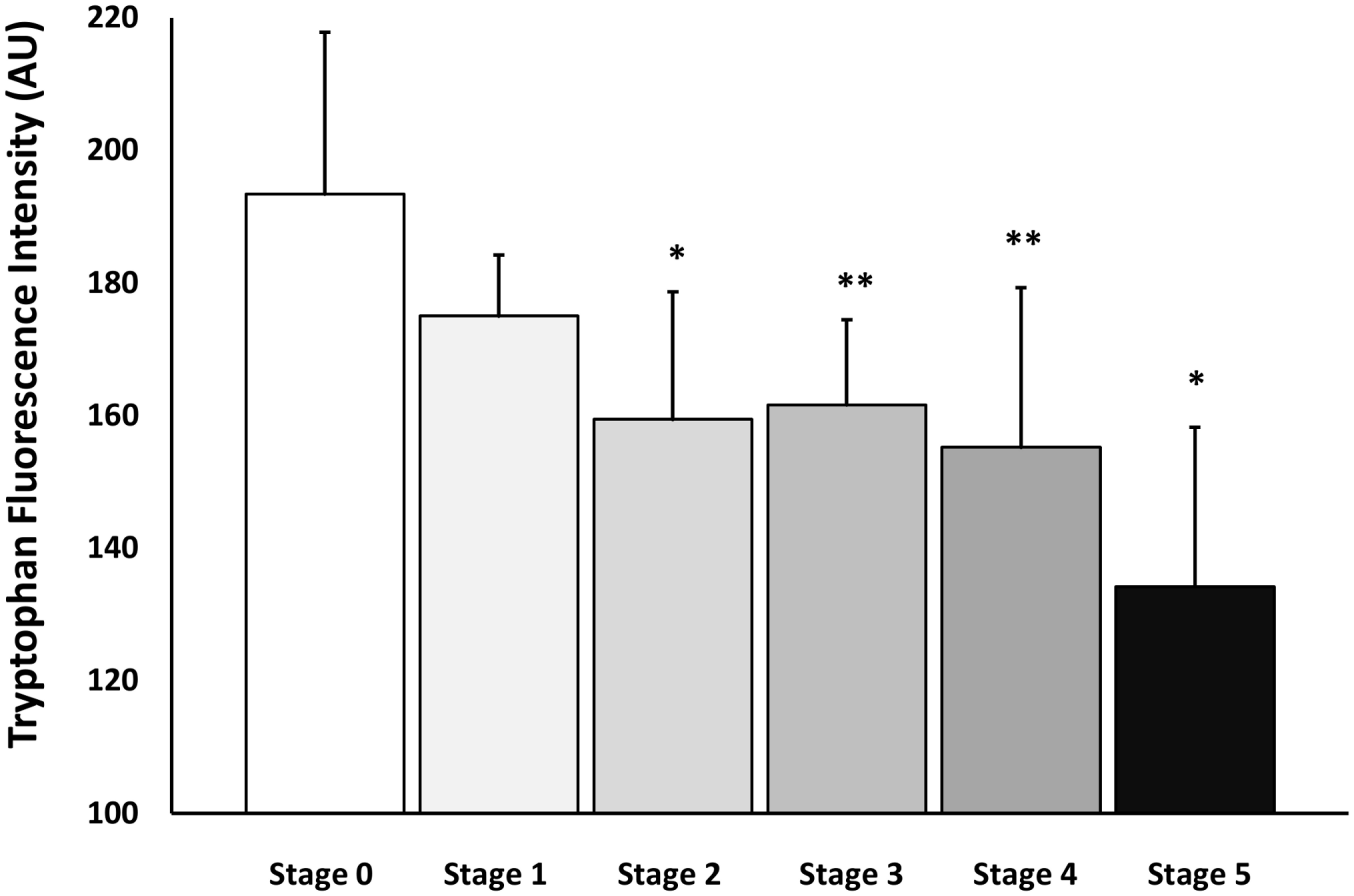
Effect of the progression of diabetic nephropathy on tryptophan fluorescence of albumin samples isolated from diabetic patients. The stages were determined from the estimated glomerular filtration rate as described in the Materials and Methods. A progressive reduction of the fluorescence intensity was observed compared to the Stage 0 control group. Values shown are mean + SD of the fluorescence of the total samples of each group. *p<0.05 vs. Stage 0 group; **p<.01 vs. Stage 0 group.

The intrinsic fluorescence of the HSA tryptophan residue was used to monitor changes in its molecular conformation. Fluorescence of albumin samples was measured using a LS 45 fluorescence spectrophotometer (Perkin-Elmer, Llantrisant, UK) with excitation and emission wavelengths of 280 and 340 nm, respectively, as described in the Materials and Methods. The differences in the fluorescence between albumin samples from controls and patients at different stages of renal disease are shown in Figure 5. The results indicate a progressive decrease in tryptophan fluorescence with the change in the estimated GFR from Stages 1–5; significant differences were achieved in Stages 2–5 compared to the Stage 0 group (p<0.001). The gradual decrease in the fluorescence emission intensity of HSA suggests conformational changes attributable to the glycation of protein that affect the environment of the residue [5], [19]. The fluorescence of HSA is dominated by tryptophan emission, and the emission spectrum of HSA is mainly due to a single residue, Trp214, in subdomain IIA [19].

**Figure 6:**
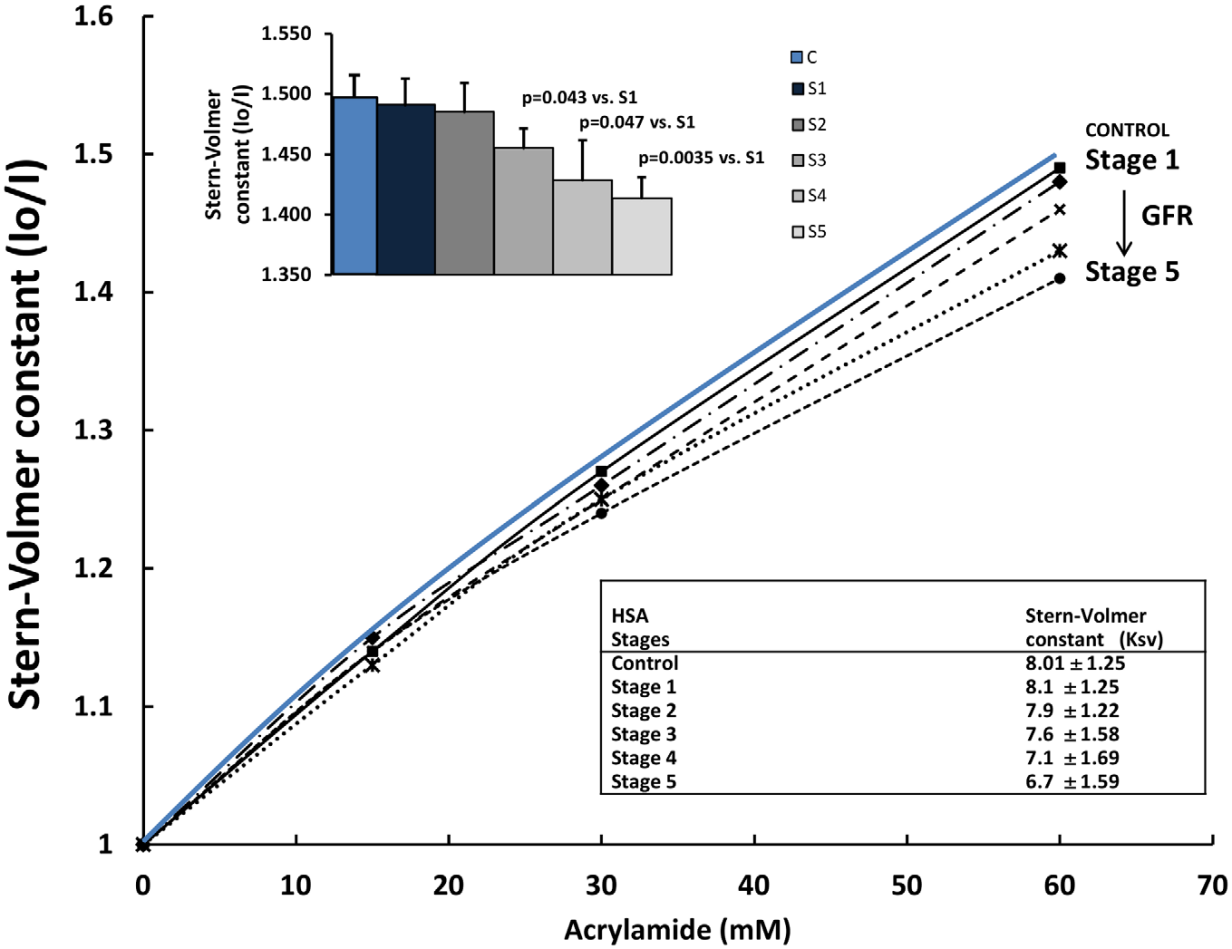
Tryptophan accessibility to the quencher in relation to the progression of diabetic nephropathy (DN). Stern-Volmer curves representing tryptophan accessibility to acrylamide reveal the existence of albumin structural changes between the advanced stages of renal disease compared with the Stage 0 control group without evidence of renal damage and a normal filtration rate. In the upper graph and table, the Stern-Volmer constants were calculated from the plot with an acrylamide concentration of 60 mM. Tryptophan fluorescence was assessed with an excitation and emission wavelengths of 280 and 340 nm, respectively. Results are mean + SD of 3 experiments with different samples from each group. Io and I correspond to the fluorescence intensities without and with the quencher, respectively. The Stern-Volmer formula for constant calculation is presented in the Materials and Methods.

The emission intensity of tryptophan on the HSA is decreased in the presence of a water-soluble quencher. The quenching by acrylamide of the fluorescence of the tryptophan residue of HSA was measured. The acrylamide Stern-Volmer plots for HSA from patients in the progressive stages of DN are shown in Figure 6 . The results indicated that the tryptophan residue of the HSA was more accessible to quenching by acylamide in the incipient stages of DN than in the advanced stages of the disease, where the tryptophan residue is inaccessible or only slightly accessible to acrylamide. The Stern-Volmer curves and the progressive reduction in the calculated Stern-Volmer constants representing the slopes of the plots suggest that conformational changes correlate with the progression of the DN. Changes in accessibility due to conformational changes have been reported [20], [21], and structural changes induced by glucose or free radicals have been demonstrated [5], [13]. Although we observed differences in the tryptophan accessibility to the quencher in DN stages, significant differences were observed only when comparing Stages 3, 4, or 5 with the Stage 0 control group (p = 0.043, p = 0.047, and p = 0.0035, respectively; Figure 6).

### Protein surface hydrophobicity

**Figure 7:**
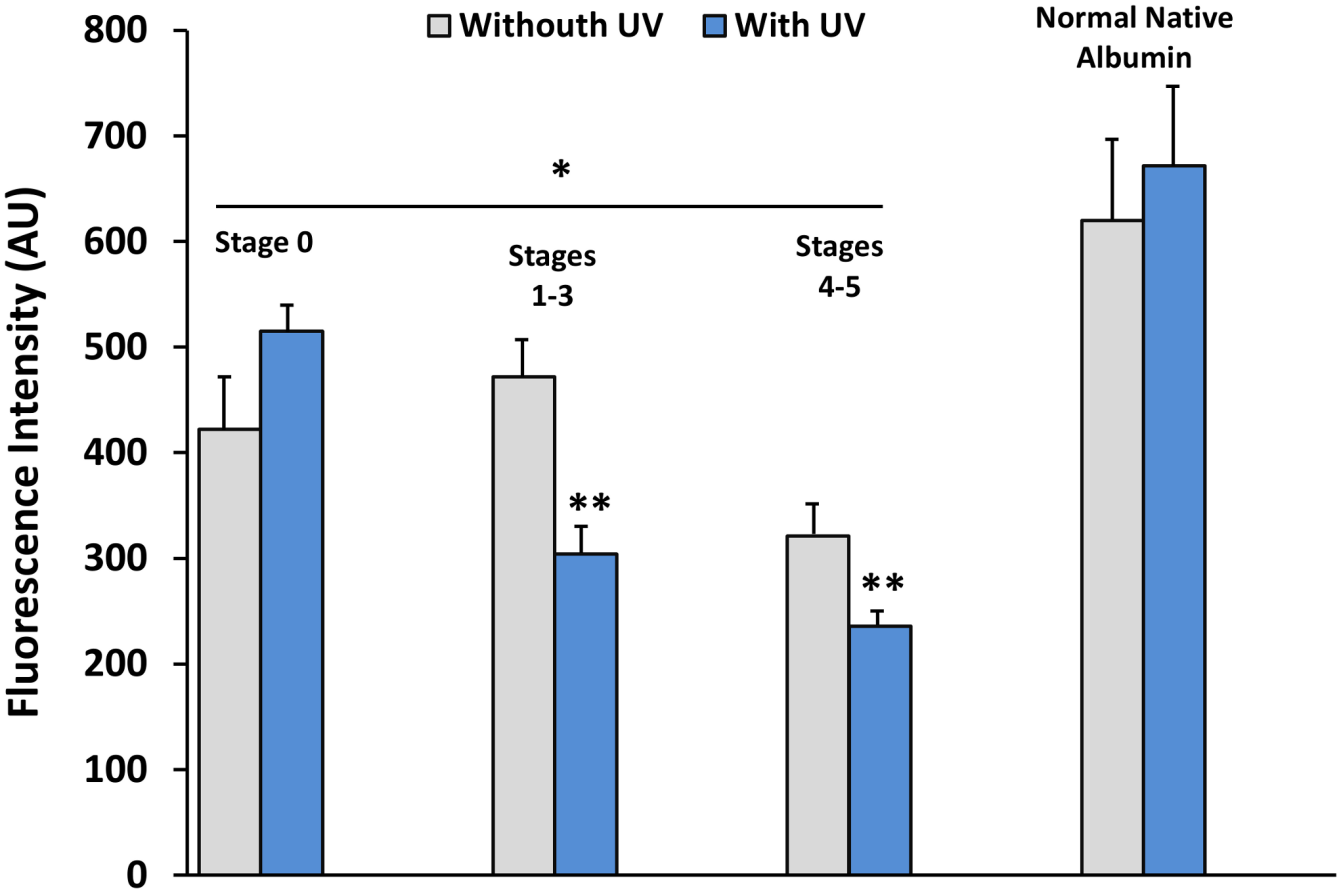
Changes in surface hydrophobicity of albumin isolated from control subjects and diabetic patients with diabetic nephropathy (DN). Changes in the surface hydrophobicity were monitored using the fluorescent probe 4,4′-dianilino-1,1′- binaphthyl -5,5′disulfonic acid (BisANS) in response to urea (3 M)-induced protein unfolding. Incorporation of the BisANS is decreased in DN patients in Stages 1–3 and decreases further in Stages 4–5 compared to the Stage 0 control group. Similarly, albumin from all groups, including the Stage 0 control group, showed lower incorporation of the fluorescent probe than native commercial albumin. Values are mean + SD of three experiments with different samples from each group. *p<0.05 vs. native albumin without UV; **p<.001 vs. Stage 0 group and native albumin with UV.

Structural alterations of proteins are generally accompanied by changes in surface hydrophobicity. In the present study, we used an adapted UV photolabeling method with the fluorescent probe 4,4′-dianilino-1, 1′- binaphthyl -5,5′-disulfonic acid (BisANS) [22] to monitor changes in the surface hydrophobic domains and changes in protein unfolding of albumin from patients in different stages of diabetic renal disease, such as described in the Materials and Methods. Incorporation of BisANS was decreased in DN patients from incipient Stages 1–3 and advanced Stages 4–5 compared with the Stage 0 control group (p<0.001; Figure 7). In fact, albumin from all of the groups, including Stage 0, showed a lower incorporation of the fluorescent probe than native commercial albumin (p<0.01). The results are consistent with our previous results [12] where commercial albumin showed higher levels of hydrophobicity and albumin modified with acrolein and partially glycated albumin quenched the level of fluorescence. A marked loss of BisANS incorporation by other proteins has also been demonstrated, particularly when exposed in vitro and in vivo to oxidative stress: a loss in the surface hydrophobic domain in creatine kinase and glyceraldehydes-3-phosphate dehydrogenase was demonstrated by the BisANS photo-incorporation before *in* *vitro* metal-catalyzed oxidation and *in* *vivo* from oxidative stress generated by rat muscle denervation [22]; the loss of hydrophobicity was accompanied by a marked reduction in activity. The changes observed in surface hydrophobicity in albumin from patients with DN can be interpreted as a disturbance in the protein structure produced by continuous exposure to the oxidative stress accompanying diabetes. An alternative or complementary explanation for this result is that hydrophobic patches in the surface of HSA were modified in advanced stages of DN as a consequence of glycation. Analyses of native and modified bovine serum albumin revealed that protein surface hydrophobicity decreases upon both albumin glycation and drug binding [23].

### Implications

The changes observed in the present work included: the RS component in albumin from DN patients increased proportionally with a reduction of the GFR; the AC of albumin from DN patients and the thiol group content tended to diminish in the advanced stages of renal disease; and a reduction in the intrinsic fluorescence and quenching of acrylamide and a reduced surface hydrophobicity, evidence of albumin conformational changes, correlated with the advanced stages of DN.

Oxidative stress is correlated with renal dysfunction in patients with renal failure [24] and plasma albumin undergoes massive oxidation in primary nephrotic syndrome [25]. Diabetic patients with DN are frequently in a state of increased oxidative stress, because free radical production is increased in diabetes. Glycation and oxidative damage cause albumin modifications, impairing its antioxidant properties [26], and producing main chain fragmentation and loss of both secondary and tertiary structure [27].

Albumin intramolecular disulfide bonds formed by the presence of Cys-Cys residues suggest that an active mechanism is linked with the sulfhydryl-disulfide turnover. The normal sulfhydryl-disulfide interchange reaction that occurs within the protein molecule leading to reorganization of the position of the disulfide-bridges was postulated several years ago [28], [29]. The Cys34 chemistry and the oxidation states, that is, reversible sulfenic (–SOH) and sulfinic (–SO_2_H), and irreversible sulfonic (–SO_3_H) acid derivatives, continue to be the prevailing explanation for the bulk of the redox potential of albumin. We postulated that an intramolecular sulfhydryl-disulfide interchange positively explains, at least partially, the RS component and the increased antioxidant potential of albumin when it undergoes structural stress. Although it has been suggested that the formation of albumin isomers in the aging process is due to thiol disulfide interchanges catalyzed by the thiol group of cysteine [30], the results presented here demonstrate an active interchange without the intervention of low molecular-weight thiols and independent of Cys34. In this respect, the possibility of the formation of multiple isomeric forms of albumin must be shown and, in fact, the formation of albumin isomers, the trapping of the intermediates free –SH groups from thiolate anions and the isolation of the isomeric forms have been reported [31]. Similarly, albumin isomerization must accompany structural alterations and aging.

**Figure 8:**
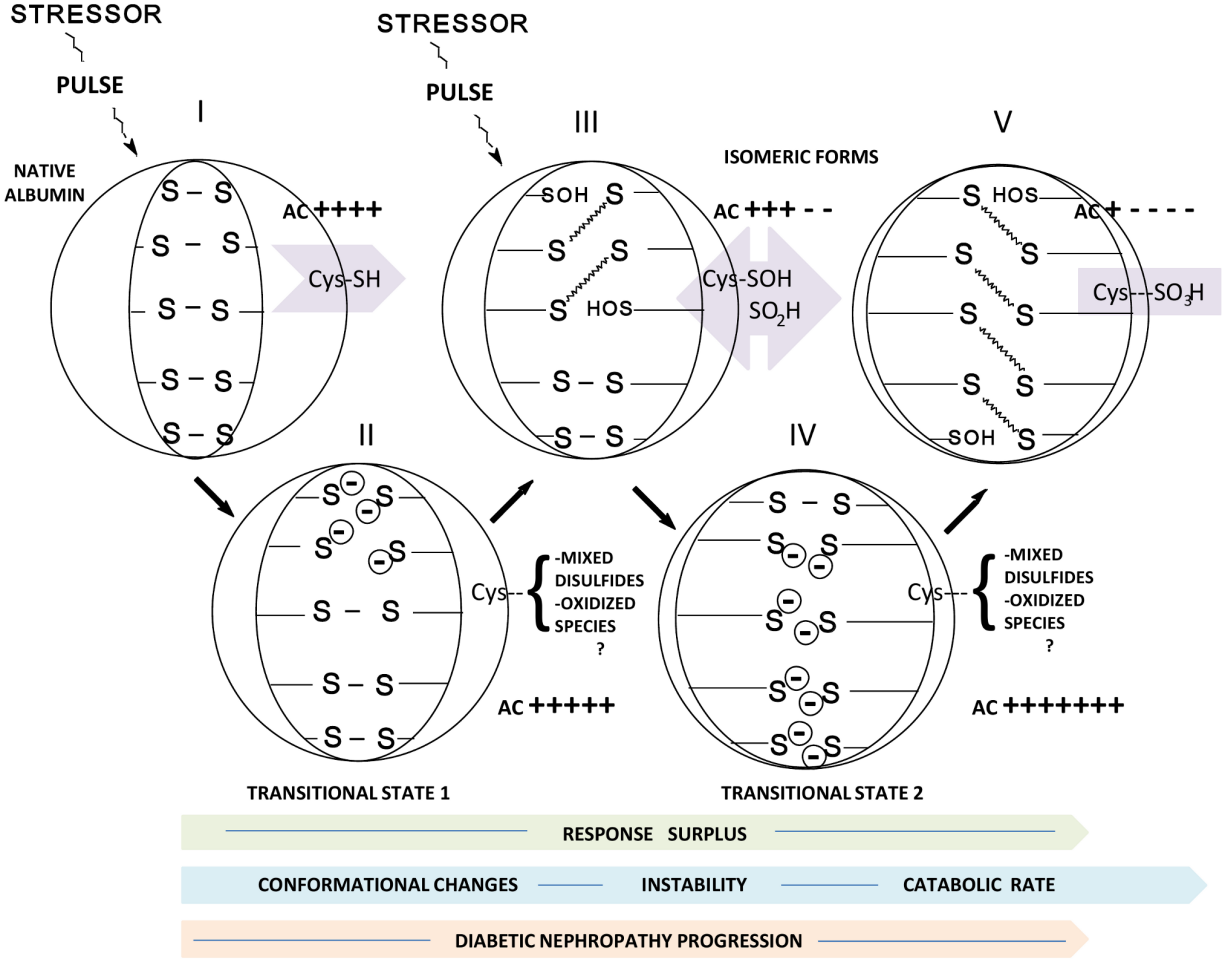
Schematic representation of the possible mechanism underlying the albumin response to stress in the progression of diabetic nephropathy. The internal region of albumin with a section of the disulfide pairing occurring between α-helices in native albumin (I) is shown. In the native state, the albumin antioxidant capacity is Cys34-dependent (violet figures). When a stressor compromises the molecular structure, cleavage of the disulfide linkage generates transition stages and the antioxidant capacity is thiolate anion-dependent (II). Transition state 1 eventually becomes an isomeric form of albumin (III). A new pulse of stress on a structurally weak isomeric form generates a second-order transition state (IV) that eventually becomes a second albumin isomeric form (V). A repetitive sequence of events results in a high response (increased Response Surplus [RS]), more and deeper structural changes, instability, and an accelerated catabolic rate (pale green, blue, and orange arrows; structural changes are represented by the shape of the internal semicircles). In general terms, isomeric forms III and V should provide low AC and the transitional states II and IV high AC. Pale violet = chemical intermediates and final product (irreversible) deriving from Cys34: thiol, sulfenic acid, sulfinic acid, and sulfonic acid (Cys-SH, Cys-SOH, Cys SO_2_H, and Cys SO_3_H, respectively). The chemical intermediates (mixed disulfides, oxidized species) of Cys34 in transition states II and IV are unknown and may depend on the specific albumin milieu. The stressor could be, for example, UV radiation, high levels or long-standing oxidative stress, high temperature, glycation, etc. AC = Antioxidant Capacity; RS = Response Surplus; Cys = Cysteine; S- = Thiolate anion.

In this regard, the modifications introduced by isomerization, including structural changes, decreased fluorescence, and aging, have consequences such as decreased resistance to catabolism and the formation of isomers with very fast catabolic rates [29], [32]. Some of these changes correlate with the results presented here and are also compatible with the mechanism proposed to explain the RS component (Figure 1). A complementary scheme represents the hypothesis of an antioxidant response to stress depending on the molecular structure, and explains the results presented here (Figure 8).

Based on our results and the scheme presented, we conclude the following:

1. The albumin AC decreases with a reduction in the filtration rate and advancing stages of DN as a consequence of the oxidation of thiol groups, derived essentially from Cys34. Under oxidative stress, the free thiol groups react, resulting in the formation of progressively more oxidized species. The formation of reversible sulfenic and sulfinic acid (HSA-SOH and HSA-SO_2_H, respectively) eventually maintains the redox state with the plasma environment at moderate, or at least not extended, exposure to oxidative stress. Evidence indicates that the HSA redox system in plasma can be activated and recover after a single bout of exercise [33]. With higher levels or extended exposure to oxidative stress, the formation of sulfonic acid (HSA-SO_3_H), the irreversible or endproduct of Cys34 SH oxidation occurs [34]. In the present work, a clear trend to a reduction in the albumin AC and reduced thiol group content together represent evidence of a progressive oxidized condition in advanced stages of DN, possibly a consequence of the continuous exposure to oxidative stress during diabetes.
2. In extreme or extended oxidative stress levels where the albumin molecular structure is compromised (structural stress), we propose transient states of transition between HSA-Cys-Cys sequences and thiolate anion (HSAS^−^) and probably the reduced moiety (HSA-SH), from the core of the protein (Figure 8). These transient intermediates with a higher AC eventually form albumin isomers. The isomeric forms of albumin could be isolated, but the transition intermediates are spontaneously transformed in a short period of time to the corresponding isomers. The albumin structure possesses an unusual disulfide-bonding pattern characterized by the eight Cys-Cys sequences (31). When albumin is stressed by short-wave UV light irradiation, the AC is increased as a response to the stress, and the cleavage of some HSA-SS groups leads to the formation of reactive thiolate anion groups (HSA-S^−^) (formation of intermediates II and IV, Figure 8). The use of a stressor *in* *vitro* reproduces in short time a phenomenon that is possibly extended *in* *vivo* with longer exposure to the stressor.
3. Transition stages are characterized by an increase in AC, but eventually all transition states result in unstable isomeric forms.
4. The increased stress response (RS) of albumin in progressively advanced stages of renal disease is likely an indirect reflection of the structural changes and instability produced by extended exposure to oxidative stress. We cannot confirm whether RS constitutes a compensatory response to oxidative threats; however, some of the albumin structural and functional consequences should include changes in the redox balance, reduced resistance to catabolism, and binding property effects of endogenous and exogenous compounds.
5. Although previous studies suggest that in extracellular fluids the albumin antioxidant activity mostly relies on the free thiol group of Cys34, the results obtained here suggest that the entire albumin molecular structure is highly involved in the antioxidant response of the protein and that the structural stress that accompanies degenerative pathologies constitutes a cumulative and measurable event.
6. Based on the present study, the RS represents an independent variable with the potential to be functional in diagnostic or prognostic possibilities for DN. For now, the changes described in point 4 above provide indirect evidence of cumulative protein instability or liability. The specific participation of the albumin conformational and functional changes in the pathogenesis of DN must be established in future studies. A specific example is the possible participation of these changes in the induction of endoplasmic reticulum stress produced in the renal proximal tubular cells by the accumulation of misfolded proteins [35], [36]. The strong association of RS with albumin structure and the analyses of the data obtained here suggest that renal function decline is related through pathways other than oxidative stress and possibly related more with albumin structural changes.

## Materials and Methods

### Chemicals

Blue sepharose was obtained from GE-HealthCare. All solutions were prepared with chemicals of pure analytical grade and filtered with a 0.45-µm filter (Millipore, Bedford, MA, USA). Horseradish peroxidase Type I (E.C. 1.11.1.1.7); hydrogen peroxide 30% (w/w); sodium HEPES (N-(2-hydroxyethyl)piperazine-N’- (2-ethanesulfonic acid) 99.5%; p-iodophenol; luminol (5-amino-2,3-dihydro-1,4-phtalazinedione); albumin from human serum (lyophilized powder, 99%, essential fatty acid free, prepared from essentially globulin free albumin; Trolox (6-hydroxy-2, 5, 7, 8-tetramethylchroman-2- carboxylic acid) were obtained from Sigma-Aldrich Inc., St. Louis, MO, USA. The centrifuge (Ultrafree-MC Centrifugal Filter device, with a 5000 and 10,000 NMWL cut-off) was obtained from AmiconMillipore Corporation.

### Patients and samples

Serum aliquots were collected from 145 institutionalized diabetic patients from the Nephrology Unit at Regional General Hospital #1, Morelia, Michoacán, México, from the hospital system of the Mexican Social Security Institute. Patients with diabetes, as assessed by American Diabetes Association criteria (fasting glucose level ≥126 mg dl^−1^ [7.0 mmol l^−1^] or 2-h glucose level ≥200 mg/dl [11.1 mmol l^−1^]) [37] were included. The exclusion criteria included a diagnosis of nondiabetic kidney disease, cancer, hypertension, supplement consumption, or medication changes during the 12-week study period.

All of the patients were informed of the study purposes and procedures and all individuals provided written informed consent prior to participation. The protocol for the research project #2010-785-061 was approved by the Ethics and Investigation National Committee (CNIC), Mexican Social Security Institute (IMSS), México. The investigation was performed in accordance with the principles of the Declaration of Helsinki and Good Clinical Practice.

The patients were divided into five groups according to their estimated GFR [38] and an additional control group (Stage 0) of early-diagnosed patients with no evidence of structural or functional renal decline. The Stage 1 group comprised patients with a normal or high GFR (between 90 and 130 mL min^−1^ 1.73 m^−2^) with history of urine or blood test abnormalities, or imaging or pathology abnormalities; Stage 2 group comprised patients with a GFR between 60 and 89 mL min^−1^ 1.73 m^−2^; Stage 3 comprised patients with a GFR between 30 and 59 mL min^−1^ 1.73 m^−2^; Stage 4 comprised patients with a GFR between 15 and 29 mL min^−1^ 1.73 m^−2^, and Stage 5 comprised patients with end-stage chronic renal failure and a GFR≤15 mL min^−1^ 1.73 m^−2^.

After an initial consultation and examination, the patients were instructed to visit the hospital Nephrology department after an 8- to 12-h overnight fast for clinical examination and sample collection; fasting blood was drawn, and serum and plasma aliquots were transferred to plastic tubes and were either used fresh or stored at –80°C for 2 to 4 weeks until analysis. Fresh samples were used for glycated hemoglobin (HbA1c), creatinine, and sulfhydryl group measurements, and albumin isolation was performed during the next 3 months in groups of six samples corresponding to each stage of DN.

### Isolation of human serum albumin

The albumin was separated and purified from 0.5-mL aliquots of human serum. The protein was purified by affinity chromatography using Blue Sepharose, packed in columns (2 mL) designed especially for this study. Before equilibration, the columns were washed with 5 bed volumes of starting buffer (50 mM citric acid, 100 mM Na_2_HPO_4_, pH 3.0). Each diluted sample was filtered with a 0.45-µm Millipore membrane, passed through the column and washed with 10 additional bed volumes of starting buffer. The albumin bound to the gel was eluted with the corresponding elution buffer (50 mM KH_2_PO_4_ and 1.5 M KCl, pH 7.0), and the albumin fraction was dialyzed in 10 mM phosphate buffer (pH 7.4), using a 3000-Dalton cut-off membrane. The protein concentration was determined and each eluted fraction was examined in 10% sodium dodecyl sulfide-polyacrylamide gels to verify protein integrity.

### Antioxidant capacity system

The AC was measured using an enhanced chemiluminescence-based assay. Horseradish peroxidase is an enzyme that catalyzes the decomposition of peroxides and forms free radicals. Peroxidase/H_2_O_2_ mixtures are used to generate free radicals [39] from several substrates. Hydrogen peroxide removes two electrons of the enzyme, and each of these is replaced in two one-electron steps, in each of which a substrate molecule forms a radical [40]. Oxygen consumption has been documented for the sodium horseradish peroxidase/hydrogen peroxide system and free radical generation [41]. Radicals formed and peroxides and oxygen oxidized luminol, producing light that could be detected and measured. Given that a constant rate of radical generation can be achieved, light emission depends on the constant production of free radicals. Enhanced chemiluminescence is used to measure the AC in biologic fluid [42]. The system used in the present work contains horseradish peroxidase (5 µU l^−1^), hydrogen peroxide 30% w/w (3.0 µM), substrate p-iodophenol (25 µM), and luminol (300 µM from a stock solution of 10 mM in DMSO). A reaction mixture containing perborate base buffer prepared with sodium tetraborate (100 mM) and sodium carbonate (100 mM) was used. Intra-assay variation for 15 samples analyzed in triplicate was 0.29%–5.2%, with a mean of 4.05%.

### Protein antioxidant capacity and protein response surplus

RS is defined as the increase in AC of a protein when it is exposed to a structural stressor [12]. To obtain RS measurements, the AC of a protein sample was determined before (ACb) and after (ACa) an oxidative or structural challenge; in the present work, UV light (254 nm) was used as the stressor. The results obtained in Trolox concentration (Trolox Equivalent Units, nM TEU) with the use of standard curves were transformed to %, considering ACb to be 100%. In this way, the values reported as RS represent the percentage of AC surplus above the mean AC of proteins before treatment. In the same way, the percent accumulated AC (%AAC) represents the sum of AC plus the RS value in percentage. To obtain RS from the results, 100 was subtracted from %AAC.

1. AC = Antioxidant Capacity (Trolox Equivalent Units, TEU)
2. %AAC, Percent Accumulated Antioxidant Capacity = (ACa) (100) ÷ ACb
3. RS%, Response Surplus % = (%AAC) −100

### Thiol group measurement

Total serum sulfhydryl groups were measured using Ellman’s reagent (5,5′-dithio-bis [2-nitrobenzoic acid)] or DTNB. The DTNB reacts with a sulfhydryl group to yield a mixed disulfide and 2-nitro-5-thiobenzoic acid, with high molar extinction coefficient at 412 nm. Samples containing 0.5 mg of purified albumin in 0.1 M sodium phosphate, pH 8.0, and 3.0 mM of EDTA were incubated for 15 min in the dark at 20°C with a DTNB solution (2.5 mM); absorbance was recorded at 412 nm, but to obtain the final value, the baseline absorbance was subtracted. Thiol values are expressed as µmol g^−1^ of albumin using a molar absorption coefficient of 14,130 M^−1^cm^−1^. A calibration curve was constructed based on the sequential dilution of 1 mmol freshly prepared cysteine stock solution at the time [43].

### Antioxidant standard preparation

Standard curves were achieved using increasing amounts of the water soluble tocopherol analog Trolox (6-hydroxy-2,5,7,8-tetramethylchroman 2-carboxylic acid). Trolox standards were prepared in milli-Q water (Millipore Corporation, Bedford, MA, USA) before initiating each experiment and starting with a stock solution of 80 µM. The AC through the experiments was expressed as nanomolar Trolox (Trolox Equivalent Units or TEU nM). Approximately 320 nM Trolox is enough to completely suppress the chemiluminescent signal and corresponds to the maximal AC achieved. One standard curve was prepared before each experiment.

### Treatment with UV light

UV light was used in the present work as a structural stressor of proteins. Isolated albumin was illuminated by a 3 UV™-36; 6 Watt, 0.16 A lamp (UVP, Upland, CA, USA) with a wavelength adjusted at 254 nm. The energy of the light used in the experiments was calculated to be 10 mW cm^−2^, monitored using a radiometer UVP model UVX-25 (UVP). The temperature for the experiments was maintained at 25±0.1°C.

### Albumin fluorescence and quenching

The presence of two tryptophan residues in albumin that generate autofluorescence allows for changes in the protein conformation to be monitored. Fluorescence measurements were performed using an LS 45 fluorescence spectrophotometer (Perkin-Elmer, Llantrisant, UK) at excitation and emission wavelengths of 280 and 340 nm, respectively. The slit width was set to 10 nm for excitation and emission. The decrease in fluorescence was analyzed according to the Stern-Volmer equation [44]:
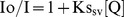
Where Io and I represent the fluorescence intensities at a wavelength in the absence and presence of the quencher, respectively, Ksv is the Stern-Volmer constant for the collisional quenching process, and [Q] is the concentration of the quencher. The quencher used in the present work was acrylamide. In the acrylamide-quenching experiments, the quencher agent was used at a final concentration of up to 60 mM, and the native albumin and isolated albumin from Stage 1–5 groups were used at a concentration of 2 g L^−1^ in phosphate-buffered saline.

### Protein hydrophobicity

Albumin hydrophobicity was measured by the incorporation of BisANS by 3 M urea treatment followed by long-wave UV light irradiation, as described previously [45] with modifications. Briefly, purified albumin samples (1 mg mL^−1^) from Stage 0 (control) and Stages 1–5 were diluted in a 3 M urea solution prepared in labeling buffer containing 50 mM Tris-HCL, 10 mM MgSO_4_ at pH 7.4. Samples were treated with 3 M urea for 2 h at room temperature. At the end, 100 mM BisANS was added and the samples agitated thoroughly with vortex. Aliquots (350 µL) were placed in a 96-well plate and incubated on ice for 1 h under exposure to long-wave UV light (365 nm) for BisANS incorporation. The samples then were placed in microcentifuge filters (10-kDa cutoff, Amicon Ultra, Millipore, Tullagreen, Carrigtwohill CO, Ireland) and washed two times with labeling buffer. Samples were re-dissolved in the same buffer and the measurement of fluorescence was performed using an LS 45 fluorescence spectrophotometer (Perkin-Elmer) at excitation and emission wavelengths of 355 and 460 nm, respectively.

### Protein sample conditioning

Before the incubation period, protein samples were placed in microcentrifuge filters (10 kDa cutoff, Amicon Ultra, Millipore) and centrifuged at 5000 g for 1 h. The filter residues were washed and dialyzed two times and dissolved in phosphate buffer (pH 7.4); the protein concentration was determined using the Bradford method [46]. Aliquots containing 100 µg of protein were irradiated with UV light for varying periods of time for each experiment and added to the reaction mixture in the luminometer cuvette after the signal was stable. The residual solvent remaining after microfiltration was collected and reacted with Folin-Ciocalteu reagent to monitor protein fragmentation. In addition, light scattering was measured at 400 nm in a spectrophotometer to check for the aggregation of irradiated proteins. The short period of albumin exposure to the UV light produced reproducible results without inducing aggregation or fragmentation. In contrast, some studies have reported structural changes and aggregation produced by UV light on lysozyme and albumin, but with very long exposure to 285-nm UV light [47].

### Statistical Analysis

Analysis of the results was performed using the statistical software SPSS 10.0 (IBM, Chicago, IL, USA). Values are expressed as mean ± SD. Groups were compared using the one-way ANOVA test followed by Dunnett’s test. Results were considered to be statistically significant when p<0.05. Pearson’s correlation coefficient (R) was used to determine the correlation between variables. The correlation was determined to be significant when the p value was less than 0.05 (5%) with Fisher’s z transformation. Multivariate models were used to assess the association between variables and the GFR decline.

## Supporting Information

Table S1 **Medications prescribed for patients included in the study from stages 0 to 5.**
(PDF)

File S1 **Database with the results of the principal variables from stages 1 to 5 and control group.** Sex, age, estimated glomerular filtration rate (GFR), glycated hemoglobin (HbA1c), blood creatinine (CREAT), antioxidant capacity (AC), Response Surplus (RS), accumulated antioxidant capacity (ACA%), thiol groups (SHs), weight, height and body mass index (BMI).
(XLSX)
