# Supplementary material for: Albumin Antioxidant Response to Stress in Diabetic Nephropathy Progression
Source: PLoS One. 2014 Sep 4;9(9):e106490. doi: 10.1371/journal.pone.0106490 (PMC4154714; doi:10.1371/journal.pone.0106490)
Supplement: Table S1 — Medications prescribed for patients included in the study from stages 0 to 5. (PDF) [file pone.0106490.s001.pdf]

Supporting information S1:

Table S1. List of miscellaneous medications prescribed for groups of patients from stage 0 to 5.

**PRESCRIPTION**	**Stage 0**	**Stage 1**	**Stage 2**	**Stage 3**	**Stage 4**	**Stage 5**
			**(%)**			
ACEI	0	30	40	30	45	20
ARB II	0	15	20	20	40	30
ARB II + ACEI	0	10	40	50	15	50
Pentoxifylline	0	55	70	80	55	20
Statins	0	20	45	65	45	15
Spironolactone	0	15	45	60	0	0
CCB	0	5	10	40	25	40
Calcium	0	0	0	55	85	95
Calcitriol	0	0	0	50	80	80
Erythropoietin	0	0	0	55	100	95
Folic acid	0	0	0	70	100	100

ACEI, angiotensin-converting enzyme inhibitor; ARB II, angiotensin II receptor blocker; CCB, calcium channel blocker.
